# The impact of the gut microbiome on memory and sleep in *Drosophila*

**DOI:** 10.1242/jeb.233619

**Published:** 2021-02-05

**Authors:** Valeria Silva, Angelina Palacios-Muñoz, Zeynep Okray, Karen L. Adair, Scott Waddell, Angela E. Douglas, John Ewer

**Affiliations:** 1 Instituto de Neurociencias, and Centro Interdisciplinario de Neurociencia de Valparaíso, Universidad de Valparaíso, Valparaíso 2360102, Chile; 2 Centro de Investigación Interoperativo en Ciencias Odontológicas y Médicas, Facultad de Odontología, Universidad de Valparaíso, Valparaíso 2360004, Chile; 3 Centre for Neural Circuits & Behaviour, University of Oxford, Oxford OX1 3TA, UK; 4 Department of Entomology, Cornell University, Ithaca, NY 14853, USA; 5 Department of Molecular Biology and Genetics, Cornell University, Ithaca, NY 14850, USA

**Keywords:** Learning, Sleep recovery, Anxiety, Circadian rhythm, Locomotor activity, Insect, Axenic, Behavior

## Abstract

The gut microbiome has been proposed to influence diverse behavioral traits of animals, although the experimental evidence is limited and often contradictory. Here, we made use of the tractability of *Drosophila melanogaster* for both behavioral analyses and microbiome studies to test how elimination of microorganisms affects a number of behavioral traits. Relative to conventional flies (i.e. with unaltered microbiome), microbiologically sterile (axenic) flies displayed a moderate reduction in memory performance in olfactory appetitive conditioning and courtship assays. The microbiological status of the flies had a small or no effect on anxiety-like behavior (centrophobism) or circadian rhythmicity of locomotor activity, but axenic flies tended to sleep for longer and displayed reduced sleep rebound after sleep deprivation. These last two effects were robust for most tests conducted on both wild-type Canton S and *w^1118^* strains, as well for tests using an isogenized panel of flies with mutations in the *period* gene, which causes altered circadian rhythmicity. Interestingly, the effect of absence of microbiota on a few behavioral features, most notably instantaneous locomotor activity speed, varied among wild-type strains. Taken together, our findings demonstrate that the microbiome can have subtle but significant effects on specific aspects of *Drosophila* behavior, some of which are dependent on genetic background.

## Abstract (precis)

**Highlighted Article:** *Drosophila* experimentally deprived of their gut microbiome display changes in learning and memory, and in the duration of sleep under standard conditions and following sleep deprivation.

## INTRODUCTION

Most animals bear microorganisms that influence their health and fitness, and the microorganisms tend to be harbored predominantly in the lumen of the gut (Engel and Moran, 2013; McFall-Ngai et al., 2013; Sender et al., 2016). These microbial communities are collectively known as the gut microbiome. There is now abundant evidence that the presence and composition of the gut microbiome can have profound effects on animal traits, particularly on metabolic and immune functions (Belkaid and Hand, 2014; Liberti and Engel, 2020; Rooks and Garrett, 2016; Van Treuren and Dodd, 2020; Visconti et al., 2019). In addition, the microbiome has been implicated, across different animals including humans, as a modulator of complex behavioral traits such as learning and memory, aspects of social behavior and mood (Vuong et al., 2017). However, the evidence for behavioral effects of the gut microbiome can be inconsistent, and the underlying mechanisms are uncertain (Forsythe et al., 2016; Johnson and Foster, 2018).

It is increasingly recognized that the traditional model animals, particularly the laboratory mouse, zebrafish, *Drosophila* and *Caenorhabditis elegans*, are excellent systems to investigate the fundamentals of animal–gut microbiome interactions (Douglas, 2019). The particular advantages of *Drosophila* for the study of behavioral effects are the wealth of quantitative assays of behavior (Zhang et al., 2010) and the ease with which microbe-free flies can be generated and maintained (Koyle et al., 2016). In the *Drosophila* literature, microbe-free flies are referred to as ‘axenic’, as distinct from ‘conventional’ flies with an unmanipulated gut microbiome.

The purpose of this study was to investigate microbiome effects on key behavioral traits of *Drosophila*: first, learning and memory, which to our knowledge has not previously been investigated from a microbiome perspective in *Drosophila*; and, second, the circadian rhythmicity of adult locomotor activity and sleep. The latter experiments build on two published studies. Schretter et al. (2018) reported that axenic *Drosophila* are hyperactive, and that specific gut bacteria can reduce locomotor activity via mechanisms involving the reduced activity of octopaminergic neurons. Selkrig et al. (2018) analyzed a number of additional behaviors including anxiety, sleep and courtship, and extended the analyses to two generations. They observed a modest increase in daytime activity and reduced night-time activity of axenic flies in the first generation, and an increase in both daytime and night-time activity of axenic flies in the second generation, and no consistent changes in the other behaviors examined.

Our study builds on this prior work by examining behaviors that might be more sensitive to the microbiota, and by using isogenic strains of flies for some assays as a means of detecting small quantitative effects. We found that the absence of the gut microbiome had a moderate effect on learning and memory, and also impacted sleep duration and recovery after sleep deprivation. We also confirmed the findings of Selkrig et al. (2018), reporting that removal of the microbiota did not affect the flies' anxiety-like behavior and locomotor activity; a similar lack of effect was observed here for circadian rhythmicity. In some cases, the lack of microbiome differentially affected the behavior of different wild-type fly strains. Differences in genetic background may explain inconsistencies in the literature on the influence of the microbiome on *Drosophila* behavior.

## MATERIALS AND METHODS

### Fly rearing and stocks

Olfactory learning and memory experiments were conducted in S.W.’s lab using a wild-type *Drosophila* strain, Canton S (CS), originating from William Quinn's laboratory (Massachusetts Institute of Technology, Cambridge, MA, USA), and were reared at 25°C, on cornmeal medium (100 g l^−1^ anhydrous d-glucose, 47.27 g l^−1^ organic maize flour, 25 g l^−1^ autolyzed yeast, 7.18 g l^−1^ agar, 12.18 g Tegosept dissolved in 8.36 ml absolute ethanol, per liter of fly food).

All other behavioral tests were carried out in J.E.’s lab. The following strains and mutants were used: CS, *yw* and *w^1118^*, which were obtained from the Bloomington *Drosophila* Stock Center (Bloomington, IN, USA); flies with mutant *period* (*per*) alleles were provided by Jeff Hall (Brandeis University, Waltham, MA, USA; *per^+^* and *per^01^*) and Patrick Emery (University of Massachusetts Medical School, Worcester, MA, USA; *per^S^* and *per^L^*), and were isogenized by crossing to *yw* flies for 10 generations. (The *white* and *period* genes are adjacent on the X-chromosome; thus, these two genes almost invariably co-segregate.) Two lines (referred to here as A and B) were isogenized in parallel for each *per* allele, to control for effects that did not map to *per* (their genotype was confirmed using locomotor activity testing at the end of the isogenization process). Results obtained using the A and B lines were comparable (Figs S1 and S2) and the data were pooled. For these assays, flies were raised at room temperature (20–22°C) under a 12 h:12 h light:dark cycle on cornmeal medium (40 g l^−1^ d-glucose, 100 ml l^−1^ cornmeal, 60 g l^−1^ fresh yeast, 7.4 g l^−1^ agar, 1.2 g l^−1^ Tegosept dissolved in 12 ml absolute ethanol).

### Generation of axenic flies

#### Generating axenic flies for olfactory learning and memory experiments (S.W. lab)

Axenic flies were produced as described in Koyle et al. (2016). For this, 0–20 h old eggs were collected on agar/apple juice plates, transferred to a cell strainer, and submerged in 3–6% sodium hypochlorite for 3–5 min until visibly dechorionated. The embryos were then rinsed multiple times with deionized sterile water. Dechorionated embryos were aseptically transferred into sterile bottles of fly food and raised at 25°C, 40–50% humidity. Axenic flies reliably presented a 36–48 h developmental delay. This delay was used as the criterion for the axenic condition because the large sample sizes needed for olfactory learning and memory experiments precluded the possibility of performing more detailed testing. Thus, any culture exhibiting less than 2 days of developmental delay was discarded (ca. 5% of total). Conventional flies were produced using the same protocol, without the dechorionation of embryos and other measures added for sterility. We obtained 200–300 flies from each bottle, a number that did not differ visibly between axenic and conventionally reared flies. To account for their slower development, axenic eggs were generated 2 days prior to conventional eggs.

#### Generating axenic flies for all other assays (J.E. lab)

Axenic flies used for all other assays were also produced as described in Koyle et al. (2016). Briefly, 0–20 h old eggs were collected on agar/apple juice plates, dechorionated for 5 min with 0.6% sodium hypochlorite, and rinsed 3 times with deionized sterile water. Approximately 60 embryos were transferred aseptically to each vial of sterilized food and raised under sterile conditions. Sterility was determined before performing any assay by homogenizing two flies from each vial in 100 µl sterile water, plating 20 µl on mMRS agar plates (Newell and Douglas, 2014), and confirming the absence of colonies after 48 h at 37°C. (We also observed a ca. 2 day developmental delay for these cultures.) Flies emerging from cultures determined to be non-axenic were discarded (10–15% of samples). Any aging of axenic flies required by an assay (e.g. courtship assays, see below) was carried out in sterilized food vials. For assays lasting several days, such as for locomotor activity and sleep, sterility was also evaluated at the end of the experiment, and results obtained from flies that were no longer sterile at the end of the experiment were discarded.

### Olfactory learning and memory experiments

#### T-maze assay

Appetitive and aversive olfactory memory were assayed using a standard T-maze paradigm, as originally described by Tully and Quinn (1985) and Tempel et al. (1983), and modified in Krashes and Waddell (2008) and Perisse et al. (2016). For appetitive memory experiments, 5–8 day old male and female flies were transferred under aseptic conditions to sterile vials with 1% agar and filter paper (approximately 150–200 flies were aliquoted per vial). The flies were kept in these vials for 24–26 h to induce starvation before olfactory training. A similar procedure was followed to prepare flies for aversive memory experiments except that they were not starved prior to testing and were instead kept in sterile vials containing fly food.

Using the T-maze setup, groups of approximately 100 flies were trained to associate an odor with an appetitive (dry sucrose) or an aversive (90 V electric shock) reinforcer depending on the experiment.

For appetitive olfactory conditioning, flies were exposed for 2 min to one odor (conditioned stimulus minus, CS−), 30 s of clean air, followed by 2 min of another odor paired with dry sucrose (conditioned stimulus plus, CS+). For aversive olfactory conditioning, flies were exposed for 1 min to an odor paired with twelve 90 V electric shocks delivered at 5 s intervals (CS+), 45 s of clean air, followed by 1 min of another odor (CS−) without shock.

To test olfactory memory, flies were given 2 min to choose between the CS− and CS+ odors in the T-maze, and were then trapped in either side and collected for counting. For immediate appetitive and aversive memory, flies were tested directly after training. For 24 h appetitive memory, flies were transferred after training into sterile food vials for 30 min of feeding, after which they were transferred back into fresh, aseptically prepared vials with agar. The flies remained in the agar vials for 24–26 h until testing. All behavior experiments were performed in temperature- and humidity-controlled chambers (23°C and 55%–65% relative humidity). Odors used in all experiments were 4-methylcyclohexanol (MCH) and 3-octanol (OCT) diluted in mineral oil to an odor dilution of ∼1:103 (specifically, 9 μl MCH, 7 μl OCT in 8 ml mineral oil).

Performance index (PI) was calculated as the number of flies in the CS+ side minus the number in the CS− side, divided by the total number of flies. A single PI data point, or *n*, represents the average PI from 2 groups of flies (each comprising approximately 100 flies), trained reciprocally on two different mazes, i.e. where the odor paired with the reinforcer was swapped between experiments.

#### Courtship-conditioning assay

The basis of the courtship-conditioning learning and memory assay is that male flies eventually stop courting a mated (unreceptive) female. This learned behavior also reduces the male fly's subsequent courtship towards a virgin female; the persistence of this reduced courtship can be used as an index of short-term and long-term memory. Courtship conditioning was carried out as described in Ejima and Griffith (2011). For these tests, virgin *per^+^* males (wild-type, from an isogenized panel of strains) were collected on the day of emergence and aged individually for 4–7 days in food vials kept at 25°C under a 12 h:12 h light:dark cycle. These test males were then exposed to a mated female for 1 h, and the courtship index (CI; proportion of time spent by the male in active courtship during 10 min or until copulation, whichever happens first) measured during the first and last 10 min of the assay, producing an initial and a final CI, respectively. The male was then transferred to a new chamber and his courtship towards a (decapitated) virgin female was recorded for 10 min (short-term memory) and after 24 h (long-term memory). A sham-trained control consisted of a virgin male placed alone in a courtship chamber for 60 min, then placed in a new chamber and his courtship towards a decapitated virgin female measured for 10 min. The various CIs obtained were then used to calculate: the learning index, which is the ratio of initial to final CI (of the test male); the CI of the test and the sham control towards the decapitated female; and the memory index, which is the ratio of the CI for the test versus control male. Thus, for the learning and memory indices, the lower the value, the greater the level of learning and memory.

### Anxiety-like behavior

Anxiety-like centrophobic behavior was assessed using an open field assay as described in Besson and Martin (2005). Briefly, *per^+^* flies (wild-type, from an isogenized panel of strains) were placed individually in a custom-made arena (4×4 cm and 3.5 mm high), and their behavior recorded during 10 min. ANY-maze tracking software (http://www.anymaze.co.uk/) was then used to measure the total distance traveled, the number of entries to the central zone of the arena, and the time spent in the center versus the periphery.

### Locomotor activity levels

Five to 10 day old males were collected on ice or under CO_2_ anesthesia, placed in *Drosophila* activity monitors (Trikinetics, Waltham, MA, USA), and their locomotor activity (detected as infrared beam crossings) recorded at 25°C under a 12 h:12 h light:dark regime. From these records, total activity during the day and night, and activity during the morning (3 h before to 3 h after lights-on) and evening peak (3 h before to 3 h after lights-off) were obtained. We also measured the instantaneous level of activity, based on the number of beam crossings per minute.

### Sleep, sleep deprivation and recovery

*Drosophila* sleep is defined as periods of 5 min or more of quiescence (Shaw et al., 2000). Sleep was measured using the same monitors used for locomotor activity except that readings were made every minute. Sleep profiles (amount of sleep per 30 min), total amount of sleep, number of sleep episodes and their length, activity per minute and total activity were derived from these readings using the MATLAB-based SCAMP software package (Donelson et al., 2012). Sleep deprivation (SD) was performed by mechanical shaking for 2 s every 10 s during the entire 12 h dark period (Liu et al., 2015) using a Troemner vortex mixer (Thorofare, West Deptford, NJ, USA). Sleep recovery after SD was determined by comparing sleep during the 24 h following sleep disruption versus the levels of baseline sleep measured during the 2 days prior to sleep disruption (Liu et al., 2015).

### Circadian rhythmicity of locomotor activity

Axenic and conventional flies were placed in *Drosophila* activity monitors, entrained for 2–4 days at 25°C under a 12 h:12 h light:dark regime, and transferred to continuous darkness for 7–10 days. Locomotor activity (number of infrared beam crossings) was recorded every 30 min. Rhythmicity was evaluated using MATLAB-based analysis software package (Levine et al., 2002). The periodicity of each record was determined using MESA (maximum entropy spectral analysis) whereas the strength of rhythmicity was obtained from the rhythmicity index (RI) derived from autocorrelation analysis, and categorized as rhythmic (RI≥0.3), weakly rhythmic (RI=0.1–0.3) or arrhythmic (RI≤0.1 and obvious aperiodic records) (Sundram et al., 2012).

### Statistical analyses

Statistical analyses were performed using GraphPad Prism (GraphPad Software, La Jolla, CA, USA). Data were analyzed using unpaired *t*-test with Welch's correction without assuming equal standard deviation. No statistical methods were used to predetermine sample size. To compare results obtained from multiple groups and/or treatments, ordinary one-way ANOVA followed by Tukey tests, or two-way ANOVA repeated measurement (RM) analyses followed by Šidák tests for multiple comparisons were performed. For assays that measured sleep duration, flies that had sleep episodes longer than 350 min or had fewer than 2 sleep episodes during the day or the night (12 h period) were discarded (<6% of the population). Additionally, Hedges' *g* corrected effect size (Hedges, 1981) was determined for all the experiments, and letters ‘S’, ‘M’ and ‘L’ were added to the figures to indicate small, medium and large effects, respectively. *P*-values and values of Hedges' *g* corrected effect size were tabulated for all results in Table S1.

## RESULTS

### Learning and memory

**Fig. 1.**
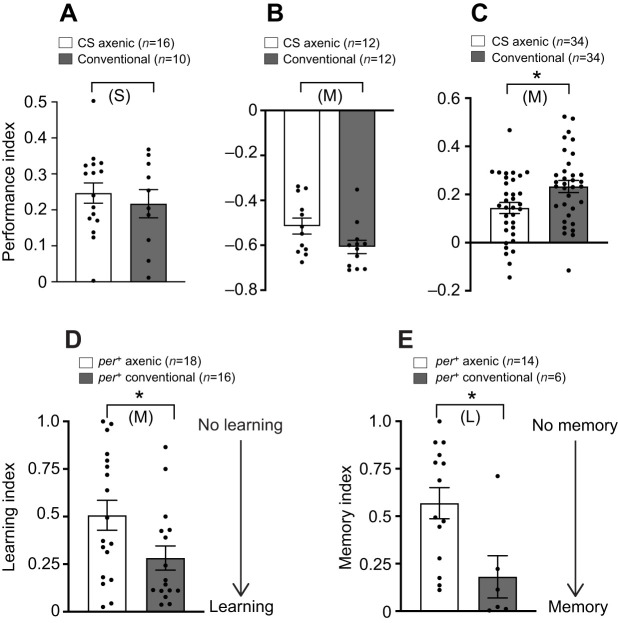
**Effect of the microbiome on learning and memory.** (A–C) Quantification of learning and memory using appetitive (A: immediate; C: after 24 h) and aversive (B: immediate) learning and memory assays for the Canton S (CS) strain of *Drosophila melanogaster*. (D,E) Quantification of learning (D) and memory (E) in a courtship-conditioning assay. For results shown in A–C, CS flies from S.W.’s lab were used; *n* corresponds to the number of T-maze iterations (∼200 flies per iteration, see Materials and Methods). For D and E, *per^+^* (wild-type) flies from an isogenic panel from J.E.’s lab were used; *n* corresponds to the number of flies tested. Results are shown as means±s.e.m. and were analyzed by unpaired *t*-test; *P*-values obtained were (A) *P*=0.5334, (B) *P*=0.0585, (C) *P<0.0107, (D) *P<0.0337 and (E) *P<0.0117; S, M, L: small, medium and large effect size, respectively; Hedges' *g* effect size analysis. See Table S1 for values.

Our first experiments investigated the effects of eliminating the microbiome on learning and memory of *Drosophila* using a standard T-maze to test for associative learning between an odor and an appetitive (sucrose) or an aversive (electric shock) stimulus. Analysis of the appetitive (Fig. 1A) and aversive (Fig. 1B) memory of conventional and axenic flies (i.e. harboring and lacking microorganisms, respectively) revealed small effects. Immediate aversive memory scores of axenic flies were moderately reduced by effect size analysis (Fig. 1B; M effect size), but not by parametric statistical analysis (*t*-test). Axenic flies expressed reduced appetitive memory at 24 h by both effect size analysis and parametric statistical tests (Fig. 1C).

As a complementary approach to test for learning and memory, we used courtship conditioning. A male fly learns that a previously mated female will reject his advances, gradually reducing his courtship towards her (Ejima and Griffith, 2011), and he then remembers this rejection, expressing less vigorous courtship towards a virgin female. In this assay, axenic males showed reduced learning (Fig. 1D) and memory (Fig. 1E) by both effects size analysis and parametric statistical analysis relative to conventional flies, indicating that elimination of the microbiome can affect learning and memory (although the relevant indices were within the range that is considered ‘normal’ learning and memory, respectively).

### Anxiety-like behavior

**Fig. 2.**
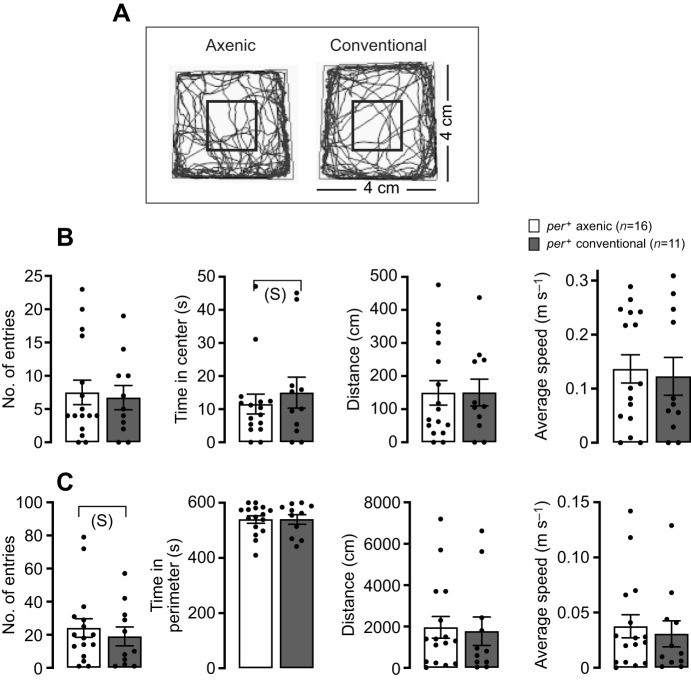
**Effect of the microbiome on anxiety-like behavior.** (A) Traces of single axenic (left) and conventional (right) wild-type flies recorded for 10 min in an arena. (B,C) Quantification of entries into the center (B; indicated by a black square in the center of the arena shown in A) and the periphery (C; corresponding to the rest of the arena shown in A) of the arena for axenic (white bars) and conventional (gray bars) wild-type flies (flies from the isogenic *per^+^* strain were used). Results are shown as means± s.e.m.; *n* corresponds to the number of flies tested. *P*>0.05 for all comparisons; S: small effect; Hedges' *g* effect size analysis; no letter means no effect. See Table S1 for values.

To investigate anxiety-like behavior in *Drosophila*, we adapted the standard centrophobism/wall-hugging assay, which relies on the tendency of anxious animals to spend proportionately less time in the central region of an open arena. The response of both male (Fig. 2) and female (data not shown) flies did not differ between axenic and conventional flies. Our data are consistent with those reported previously by Selkrig et al. (2018).

### Intensity and circadian rhythmicity of locomotor activity

We next examined the consequences of eliminating the microbiome on the intensity and circadian rhythmicity of locomotor activity. The influence of the microbiome on locomotor speed has previously been investigated (Selkrig et al., 2018; Schretter et al., 2018), with contradictory results. However, to our knowledge, the effect of the microbiome on circadian rhythmicity of locomotor activity has not been studied, although it is a highly quantifiable trait. We hypothesized that circadian rhythmicity phenotypes may be further sensitized by the elimination of the microbiome if the flies carried mutations, such as the *per*^*L*^ *per* allele, which makes their phenotype less robust. The *per^L^* allele causes the expression of a long periodicity of circadian rhythmicity as a result of hypomorphic gene function, which renders its clock especially sensitive to temperature (Konopka et al., 1989; Ewer et al., 1990). Focusing on the *per* locus is also advantageous because it includes an allelic series that spans from very strong (*per^S^*) to wild-type (*per^+^*), weak (*per^L^*) and absent (*per^01^*) rhythmicity. Therefore, for our analysis of locomotor activity, we used flies bearing each one of these four *period* alleles (*per^+^*, *per^S^*, *per^L^* or *per^01^*), all of which had recently been made isogenic through repeated backcrosses to a common *yw* laboratory strain. This analysis also included non-isogenic laboratory strains of (*per^+^*) CS and *w^1118^* flies.

**Fig. 3.**
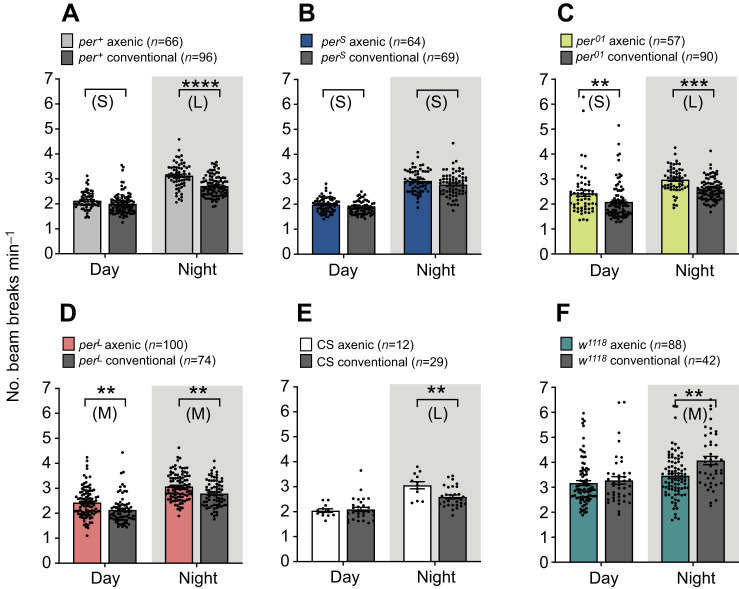
**Effect of the microbiome on speed of locomotor activity.** Mean (±s.e.m.) number of beam breaks per minute during the day and night under 12 h:12 h light:dark conditions, averaged over 2 days, for axenic and conventional flies. Results for axenic flies are colored as indicated; corresponding results for conventional flies are indicated in gray regardless of genotype. *n* indicates the number of flies tested. Data were analyzed by a two-way RM ANOVA and Šidák test where **P<0.0021, ***P<0.0002 and ****P<0.0001. S, M, L: small, medium and large effect size, respectively; Hedges' *g* effect size analysis; no letter or symbol means no effect. See Table S1 for values.

**Fig. 4.**
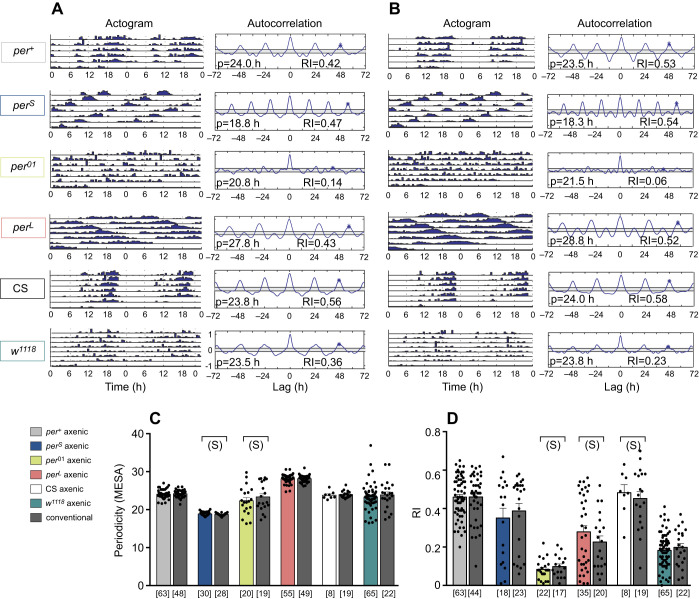
**Effect of the microbiome on the circadian rhythm of adult locomotor activity.** (A,B) Actogram (left panels) and autocorrelogram (right panels; periodicity, p, and rhythmicity index, RI, indicated within each panel) for individual axenic (A) and conventional (non-axenic; B) adult flies under conditions of constant darkness. (C,D) Corresponding mean (±s.e.m.) periodicity obtained from MESA analyses (C), and RI (±s.e.m.) obtained from autocorrelation analyses (D). In A and B, flies used for results shown in the top 4 records (*per^+^*, *per^S^*, *per^01^* and *per^L^*) are from isogenic strains; CS and *w^1118^* are non-isogenic controls. In C and D, each point represents a different individual. Genotypes are color coded for axenic flies as indicated in the key; these results are paired with those for corresponding conventional flies colored in gray regardless of genotype. For all genotypes, *P*>0.05 for all comparisons between axenic and conventional flies (Welch's *t*-test); S: small effect size; Hedges' *g* effect size analysis; no letter means no effect. See Table S1 for values. The number of flies tested is indicated below each column in brackets.

In the first experiments, the locomotor activity of axenic and conventional flies was evaluated over 2 days under a 12 h:12 h light:dark regime (Fig. 3). Instantaneous activity (measured as the number of beam crossings per unit time) was elevated for three genotypes (*per^+^*, *per^01^* and *per^L^*) and for CS flies during the night; and, additionally, for *per^01^* and *per^L^* flies during the day (Fig. 3A–E). Exceptionally, *w^1118^* flies displayed the reverse effect of reduced night-time locomotor activity in axenic flies (Fig. 3F). As these flies carry the wild-type (*per^+^*) allele (see Fig. 4, below), this result reveals that the consequences of being germ-free can also depend on genetic background.

As an alternative approach to assess the instantaneous speed of locomotor activity, we calculated the speed of movement of *per^+^* flies in the wall-following assay. Whether in the open arena or at the periphery, the average speed of axenic flies tended to be higher than that of conventional flies, but the effect was not statistically supported with the sample size used (Fig. 2B,C).

We then analyzed the circadian rhythmicity of locomotor activity by monitoring the pattern of activity (measured as the number of beam breaks per 30 min) of axenic and conventional flies for 7–10 days in constant darkness. All flies expressed the expected periodicities and rhythmicity for their genotype (Fig. 4). Furthermore, this periodicity (Fig. 4C) and its strength (Fig. 4D) did not differ in conventional versus corresponding axenic flies. Thus, our results show that circadian rhythmicity is not affected by the lack of microbiome even when flies carry the hypomorphic *per^L^* allele.

To determine whether the microbiota affected the pattern of activity during the day, we evaluated the distribution of activity during 2 days under a 12 h:12 h light:dark regime for axenic and conventional flies. When considering the profiles of activity, all axenic and conventional flies behaved qualitatively according to their *per* genotype (Fig. S3A–F), with all *per^+^* flies (*per^+^* from isogenic strain, CS and *w^1118^*; Fig. S3A,E,F) showing increased activity during the early part of the day (morning peak) and a prominent evening peak, which anticipated lights-off. This evening peak was advanced and delayed for *per^S^* (Fig. S3B) and *per^L^* (Fig. S3D) flies, respectively, and absent in *per^01^* flies (Fig. S3C), for which the evening peak is a startle response to lights-off. Quantitative analyses of the results shown in Fig. S3 revealed subtle differences for some genotypes between conventional and axenic flies in terms of the total day and night activity (Fig. S4A–F) and the amplitude of morning or evening peaks of activity (Fig. S4G–L). Interestingly, although some differences were observed with regard to the effect of the microbiota on the various isogenic *per* alleles tested, the impact of the microbiota could again also differ among flies of the same *per* genotype but different genetic backgrounds. Thus, for example, whereas axenic *per^+^* flies from the isogenized line were less active during the morning peak of activity than their conventional control (Fig. S4G), this difference was not observed for axenic versus conventional CS flies (which are also *per^+^*) (Fig. S4K).

### Sleep behavior

**Fig. 5.**
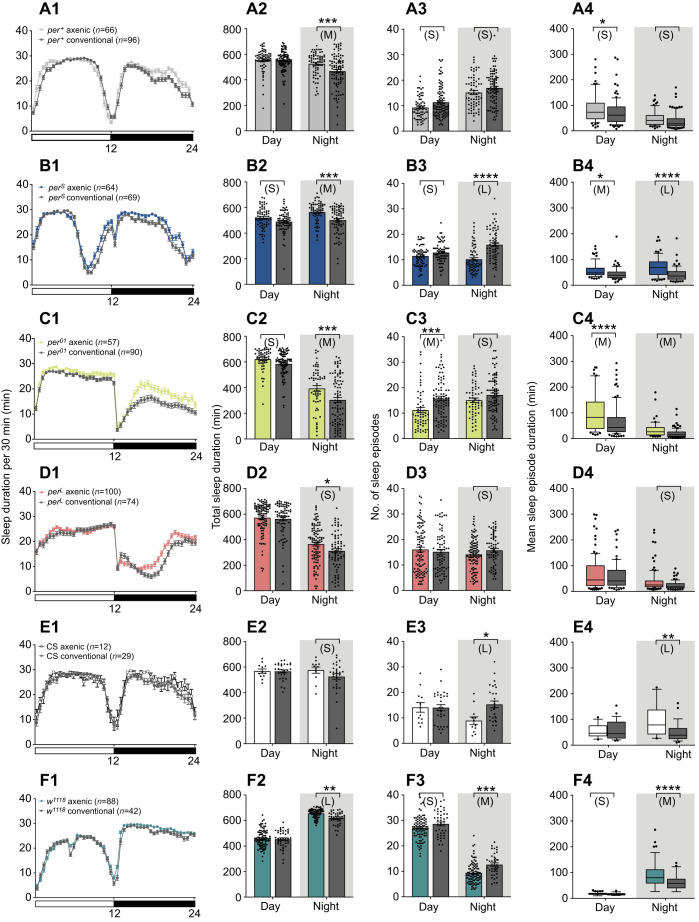
**Effect of the microbiome on sleep and sleep consolidation.** Average sleep duration per 30 min (A1–F1), total amount of sleep (A2–F2), number of sleep episodes (A3–F3) and sleep episode duration (A4–F4), averaged over 2 days (means±s.e.m.) under 12 h:12 h light:dark conditions, for axenic and conventional flies. Results for axenic flies are colored as indicated; corresponding results for conventional flies are indicated using a gray line and fill regardless of genotype. Flies used for results shown in A–D (*per^+^*, *per^S^*, *per^01^* and *per^L^*) are from isogenic strains and those in E and F are non-isogenic controls (CS and *w^1118^*). The number of flies used for each genotype and condition is indicated in A1–F1. Data were analyzed by a two-way RM ANOVA and Šidák test where *P<0.0332, **P<0.0021, ***P<0.0002 and ****P<0.0001. (Results for isogenic sub-lines A and B are shown separately in Fig. S2.) S, M, L: small, medium and large effect size, respectively; Hedges' *g* effect size analysis; no letter or symbol means no effect. See Table S1 for values.

Further analysis of the datasets obtained for flies maintained under the 12 h:12 h light:dark regime revealed that axenic flies of all isogenic *per* alleles slept more at night (longer total night-time sleep duration) than did their corresponding conventional controls (Fig. 5A2–D2). A similar effect was observed for *w^1118^* flies, and the results for the CS strain trended in the same direction, although differences were marginal.

**Fig. 6.**
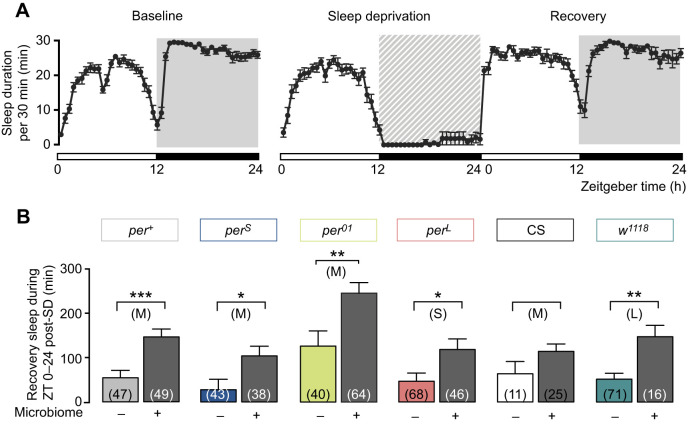
**Effect of the microbiome on sleep recovery.** (A) Example sleep profile (obtained from conventional *w^1118^* flies) of mean (±s.e.m.) sleep: during the 2 days prior to sleep deprivation (left; mean of 2 days is shown); during the day of night-time sleep deprivation (middle); and during day following sleep deprivation. (B) Quantification of mean (±s.e.m.) sleep recovery during the entire day (Zeitgeber time, ZT; 24 h) after sleep deprivation (SD) in axenic and corresponding conventional flies. Results for axenic flies are colored as indicated; corresponding results for conventional flies are indicated in gray regardless of genotype. Flies used for *per^+^*, *per^S^*, *per^01^* and *per^L^* genotypes are from isogenic strains. Number of flies used is indicated within histograms in B. Data were analyzed by unpaired *t*-test, where *P<0.0332, **P<0.0021 and ***P<0.0002; no asterisk means *P*>0.05; S, M, L: small, medium and large effect size, respectively; Hedges' *g* effect size analysis. See Table S1 for values.

Because axenic flies showed altered sleep duration yet expressed normal circadian rhythmicity (Fig. 4), we next investigated whether their sleep defects might be due to alterations in the homeostatic component of sleep by determining their capacity to recover lost sleep after sleep deprivation. Overall, the duration of the sleep recovered during the first 24 h after sleep deprivation was considerably reduced for axenic flies relative to conventional flies (Fig. 6B), and this effect was observed, albeit to a different extent, for all fly strains. Importantly, the amount of sleep recovered differed amongst ostensibly wild-type flies (e.g. *per^+^* versus CS, Fig. 6B). In addition, we noticed that sleep recovery occurred mostly during the night following sleep deprivation, with the exception of *w^1118^* flies, which recovered all their lost sleep in the first 12 h (not shown), again revealing an effect due to genetic background.

During sleep deprivation, flies are shaken using a vortexer (for 2 s every 10 s; see Materials and Methods) and are many times unable to move. As sleep is defined as periods of 5 min of quiescence, this lack of movement can spuriously be recorded as sleep. Curiously, however, we noticed that these periods of immobility caused by the frequent shaking sometimes differed between axenic and conventional flies of the same genotype (Fig. S5A–F). To check whether the amount of sleep recovered depended on the level of ‘sleep’ recorded during sleep deprivation, we analyzed the relationship between the level of immobility observed during sleep deprivation and the amount of sleep during the recovery period. As shown in Fig. S5, there was no clear relationship between these two parameters. For example, for *per*^+^ (Fig. S5A) and *per^01^* (Fig. S5C), conventional flies were apparently less immobilized during the sleep deprivation period than were the corresponding axenic flies, yet they showed a greater sleep rebound (Fig. S5A′,C′). By contrast, conventional *w^1118^* flies showed an apparent complete sleep deprivation (Fig. S5F), which was much greater than that for the corresponding axenic flies, yet showed a similar sleep rebound to that seen for *per^+^* and *per^01^* (Fig. S5F′). Importantly, however, conventional flies always recovered more sleep during the first 24 h after sleep deprivation than did the corresponding axenic flies, regardless of their genotype (Fig. 6; Fig. S5A′–F′).

## DISCUSSION

We know from the extensive literature on interactions between animals and pathogens that microorganisms can have a profound effect on animal behavior. Various pathogens manipulate their host, for example, to lose fear of predators or to move to exposed locations, thereby promoting pathogen transmission (Heil, 2016; Hughes, 2014). The effect of non-pathogenic microorganisms on host behavior is predicted to be more nuanced because of the greater overlap in the selective interests of host and microorganisms (Douglas, 2018b; Ezenwa et al., 2012), and a current research priority is to identify the categories of animal behavior that are influenced by the presence and composition of the microbiome and the host–microbe interactions mediating these effects (Cryan et al., 2019; Long-Smith et al., 2020; Palacios-García and Parada, 2019; Vuong et al., 2017).

This study has identified significant effects of the microbiome on, first, some aspects of learning and memory and, second, sleep homeostasis in *Drosophila*. Here, we discuss these behavioral traits in turn, in the context of the wider literature on microbiome effects on behavior.

To our knowledge, this study provides the first investigation of microbiome effects on learning and memory in *Drosophila*. We provide evidence from both sugar-rewarded olfactory conditioning and courtship conditioning that axenic flies have a moderate impairment in learning and memory. Our results, therefore, indicate that, in the absence of the microbiome, *Drosophila* might display small defects in the consolidation of nutrient-dependent long-term memory (Burke et al., 2012) and/or in the hunger-dependent expression of long-term appetitive memory (Krashes et al., 2009). Reduced memory has also been demonstrated in microbiologically sterile rodent models, together with evidence that specific bacteria, notably *Lactobacillus* species, can improve memory in both rats and mice (Mao et al., 2020; Mohammadi et al., 2016; O'Hagan et al., 2017). A priority for future research on the mechanisms of microbiota-dependent learning and memory is to develop novel experimental setups that facilitate aseptic conditions for tight control and precise manipulation of gut microbiota.

Turning to the effects of the microbiome on locomotor activity in *Drosophila*, the results of this study build on two previous publications (Schretter et al., 2018; Selkrig et al., 2018). There are both similarities and discrepancies across the three studies. Using the *Drosophila* activity monitoring system, Schretter et al. (2018) demonstrated that axenic female flies are hyperactive during the daytime, while we obtained hyperactivity of male flies in four of the six *Drosophila* strains tested, predominantly during the night, and the reversed effect for one strain, *w^1118^*. However, beyond differences in average speed, there are also significant differences in the profiles of activity shown by flies (conventional and axenic) in Schretter et al. (2018), Selkrig et al. (2018) and in our results (Fig. S3). For example, the wild-type flies in Schretter et al. (2018) do not show the characteristic increase in activity that occurs at dawn, which is quite prominent in Selkrig et al. (2018) and in our results (Fig. S3A,E,F). In addition, although in all three studies the levels of activity show the characteristic dip during the day, this behavior is less pronounced in both Selkrig et al. (2018) and Schretter et al. (2018) than in the wild-type flies used in our study (Fig. S3A,E,F). The different genetic backgrounds in the three studies may contribute to these differences, based on our observation that the behavior of axenic flies in this study depends on genetic background. In addition to locomotor activity, we examined here the influence of the microbiome on circadian rhythmicity, failing to detect an effect even when flies carried the hypomorphic *per^L^* allele. Finally, and in agreement with Selkrig et al. (2018), we found that night-time sleep duration is extended in axenic flies, with the implication that axenic flies sleep for longer but move around faster when awake during the night, relative to conventional flies. [Selkrig et al. (2018) observed a reversal of the difference in sleep duration between second generation axenic flies and conventional flies, but this comparison was not made in our study.]

In general terms, the several among-study discrepancies in microbiome effects on locomotor activity of *Drosophila* can be attributed, at least partly, to the differences in host genotype and diet, both of which are known to influence microbiome effects on host traits in *Drosophila* (Consuegra et al., 2020; Dobson et al., 2015; Jehrke et al., 2018; Keebaugh et al., 2019; Matthews et al., 2020; Wong et al., 2014) and other animals (Ezra-Nevo et al., 2020; Leigh and Morris, 2020). Two further issues relate specifically to this study. First, the distinctive results for sleep in strain *w^1118^* may be linked to the evidence that this strain, although widely used in genetic studies of *Drosophila*, is subject to metabolic and neurodegenerative lesions, causing impaired vision and mobility (Evans et al., 2008; Ferreiro et al., 2017; Sullivan et al., 1980). Second, although the microbiome can markedly influence the penetrance of some mutations (Dobson et al., 2015), our hypothesis that microbiome effects on locomotor activity and circadian rhythmicity would be amplified in *per* mutants was not supported, indicating that mutant *per* alleles do not offer a useful sensitized background for this assay.

Taking these analyses of locomotor activity together with other host traits, it is becoming increasingly clear that microbiome effects on host traits vary in robustness. While some effects are reliably repeated in different studies, e.g. microbiome-mediated promotion of larval developmental rate (Newell and Douglas, 2014; Storelli et al., 2011; Walters et al., 2020), others yield contradictory results, e.g. microbiome effects on lifespan (Brummel et al., 2004; Fast et al., 2018; Keebaugh et al., 2019; Ren et al., 2007). For locomotor activity, there is a general trend of increased activity in axenic flies, although the circadian timing and magnitude of this effect vary with assay protocol, and the genotype and possibly sex of *Drosophila* (Schretter et al., 2018; Selkrig et al., 2018; this study). Generally, this variation should be treated not as a hindrance to progress but as an opportunity to assist in deciphering the mechanism (Douglas, 2018a).

A high priority for future research is to determine the neurobiological basis of the differences in locomotor activity and sleep (including sleep recovery) between axenic and conventional *Drosophila*. Of particular interest is the role of *Drosophila* insulin-like peptides (DILPs), following evidence that, first, insulin-secreting neurons of the *pars intercerebralis* are implicated in the wake-promoting effects of octopamine (Crocker et al., 2010) and, second, that *dilp* gene expression is significantly reduced in axenic flies (Shin et al., 2011). A further productive lead is the activity of octopaminergic neurons, which have been linked with the hyperactivity of axenic flies (Schretter et al., 2018), as well as with reinforcing appetitive olfactory memory in the mushroom bodies (Burke et al., 2012; Schwaerzel et al., 2003) and reduced demand for rebound sleep following sleep deprivation (Seidner et al., 2015). Information on candidate microbial products that may mediate microbiome effects is also available, including evidence that bacteria-derived acetic acid promotes insulin signaling (Kamareddine et al., 2018; Shin et al., 2011) and that a bacterial sugar isomerase underlies microbiome effects on locomotor activity (Schretter et al., 2018).

As for all model organisms, *Drosophila* research is of greatest value where it illuminates conserved, rather than taxon-specific, biological processes. There is growing evidence for parallels in the neural circuits controlling wakefulness and activity between the *Drosophila* and mammalian brain (Anafi et al., 2019; Helfrich-Forster, 2018; Keene and Duboue, 2018), and many aspects of interactions between animals and their gut microbiome are also conserved across the animal kingdom (Douglas, 2018b; McFall-Ngai et al., 2013). We should not, however, expect exact equivalence of microbiome effects between different animals. For example, the apparent lack of microbiome effects on wall-following behavior in *Drosophila* (Selkrig et al., 2018; this study) indicates that *Drosophila* is not a suitable model to study microbiome effects on anxiety-like behavior in mammals. For the behavioral traits of activity and wake/sleep cycles, however, the genetic resources and tractable microbiome of *Drosophila* offer great opportunity for the elucidation of mechanisms underlying microbiome-dependent traits.
